# The Impact of Front‐of‐Pack Labeling on Social Inequality in Sugar Consumption and Purchase: A Systematic Review

**DOI:** 10.1111/obr.70150

**Published:** 2026-05-19

**Authors:** Ziyao Ge, Claudia H. Marck, Adyya Gupta, Gagandeep Kaur, Roisin McGrath, Ankur Singh

**Affiliations:** ^1^ Centre for Epidemiology and Biostatistics, Melbourne School of Population and Global Health The University of Melbourne Melbourne Victoria Australia; ^2^ Disability and Health Unit, Centre for Health Policy, Melbourne School of Population and Global Health The University of Melbourne Melbourne Victoria Australia; ^3^ Deakin Centre for Global Preventive Health and Nutrition, Deakin Institute for Health Transformation, School of Health and Social Development, Faculty of Health Deakin University Geelong Victoria Australia; ^4^ University of Sydney School of Dentistry The University of Sydney Sydney New South Wales Australia; ^5^ Charles Perkins Centre The University of Sydney Sydney New South Wales Australia; ^6^ Melbourne Dental School The University of Melbourne Melbourne Victoria Australia

**Keywords:** front‐of‐pack labeling, social inequality, sugar consumption, systematic review

## Abstract

**Objective:**

This systematic review examined the impact of front‐of‐pack labeling (FOPL) on social inequality in sugar consumption and purchase.

**Methods:**

We searched Ovid MEDLINE, Scopus, EMBASE, and Web of Science for studies published in English since 2005. Eligible studies included randomized controlled trials and observational studies. The screening was conducted independently by at least two reviewers. Data extraction and quality assessments were cross‐checked. The risk of bias was assessed using ROBINS‐E, RoB2, and CHEERS 2022. A narrative synthesis summarized the findings.

**Results:**

Of the total of 7701 studies identified, 10 studies were included. All included studies were conducted in high‐ and upper‐middle‐income countries, and primarily evaluated one specific type of FOPL, specifically warning labels (*n* = 8). All studies reported FOPL reduced sugar consumption at the population level. However, findings on social inequality were inconsistent. Five studies showed greater benefit among higher‐socioeconomic groups, four among lower‐socioeconomic groups, two identified a U‐shaped effect, and two found no differential effect by socioeconomic status. The overall quality of studies was poor.

**Discussion:**

FOPL, in particular warning labels, can reduce sugar consumption, but its effects on socioeconomic inequalities were inconsistent. Evaluation of high‐quality longitudinal research is needed to ensure FOPL interventions are inclusive and effective across socioeconomic groups.

**Abbreviations**
AIGC: artificial intelligence generated content
BOPL: back‐of‐pack labeling
CHEERS 2022: Consolidated Health Economic Evaluation Reporting Standards 2022
DALYs: disability‐adjusted life years
FOPL: front‐of‐pack labeling
GDA: guideline daily amount
HICs: high‐income countries
LMICs: lower‐middle‐income countries
NCDs: noncommunicable diseases
NR: not reported
PRISMA: Preferred Reporting Items for Systematic Reviews and Meta‐Analyses
RCT: randomized controlled trial
RoB 2: risk‐of‐bias tool for randomized trials
ROBINS‐E: risk of bias in non‐randomized studies—of exposures
SES: socioeconomic status
SSB: sugar‐sweetened beverage
TLS: traffic light system
UI: uncertainty interval
UK: United Kingdom
UMICs: upper‐middle‐income countries
US: United States
WHO: World Health Organization
WIC: Special Supplemental Nutrition Program for Women, Infants, and Children
WL: warning label

## 1 Introduction

High sugar consumption poses a significant public health issue, as it is associated with the risk of developing noncommunicable diseases (NCDs) including obesity, diabetes, cardiovascular diseases, dental caries, and even cancer [1]. In 2019, the global burden of disease attributable to high sugar sweetened beverages (SSB) consumption was estimated at 6,307,562 disability‐adjusted life years (DALYs) (95% CI: 4,300,765, 8,079,556) across 204 countries and territories [2]. Despite the well‐documented health risks associated with excessive sugar consumption and the World Health Organization (WHO) [3] recommendations to limit free sugar intake to less than 5%—and ideally below 10%—of total energy intake, the majority of countries worldwide are exceeding these guidelines [4].

Social inequality is defined as unequal distribution of, and unequal access to valued resources, opportunities, and social positions [5]. Social inequality in sugar consumption is evident between countries and within countries. For example, a recent study showed that SSB sales decreased by 22% in high‐income countries (HICs) but increased by 13% in upper‐middle income countries (UMICs) and by 50% in lower‐middle‐income countries (LMICs) between 2007 and 2019 [6]. Similarly, ultra‐processed foods sales grew during 2009 to 2019, with the growth in LMICs being 11 times higher than in HICs [7]. At the individual level, socioeconomic status (SES) is associated with unhealthy diet patterns, including high sugar consumption [8]. In the United States (US), children and adolescents from low‐income families, or with less educated parents, had 1.93 and 1.28 times the odds, respectively, of consuming SSB heavily than those with more educated parents [9]. A longitudinal study in Norway further supported this finding, demonstrating that social inequality in sugar consumption during childhood persists into adulthood [10]. In the United Kingdom (UK), households with low SES were more likely to purchase high‐sugar foods, which accounted for 34.8% of their total energy intake [11]. This is more than three times the WHO recommended limit of less than 10% of total energy intake per day [12]. In the UK, differences in sugar purchases between the lowest and the highest social grades were 3.9 g (95% CI: 2.9, 4.8) for table sugar [8]. These inequalities have potentially long‐term consequences, contributing to cumulative health risks across populations over time, particularly among those experiencing social disadvantage. A social gradient is evident in conditions such as dental caries [13], overweight, mental ill‐health, and their comorbidity [14]. To address the public health issue of high sugar consumption and reduce associated social inequality, structural population‐level strategies are urgently needed [15].

Front‐of‐pack labeling (FOPL), a form of food labeling, is a population‐level strategy to provide consumers with food information on the nutrient content of the food to enable healthier food choices [12, 16]. Examples of FOPL include health warning labels (WL), reference intakes, energy icons, Nutri‐Score food labels, Health Star Rating systems (HSR), and Healthy Choice Logos [17, 18]. The overarching objectives of FOPL are threefold: (1) to inform consumers to make healthier food choices, (2) to encourage manufacturers to reformulate current products or develop healthier products, and (3) to improve public health in a non‐enforcing way [19]. The WHO recommends FOPL as a key policy tool for improving diet and reducing the diet‐related disease burden [3].

FOPL is more effective than back‐of‐pack labeling (BOPL) in improving diet quality, particularly in reducing sugar consumption [20], with substantial evidence supporting its impact [21, 22, 23, 24, 25, 26, 27]. Although FOPL is proposed as an equitable policy with the observation of reduced social inequality in sugar consumption [28], findings on its effectiveness across SES subgroups are mixed, with some studies showing greater benefits among individuals with higher SES [29, 30]. Systematic reviews primarily evaluated FOPL awareness and usage, its impact on health‐related behaviors (including food choice, the healthfulness of food purchases, and purchase or consumption intentions), and the effectiveness of different FOPL types [31, 32, 33]. Kelly et al.'s review indicated FOPL improved dietary behaviors with some interpretive FOPL systems (i.e., HSR) showing a greater effect than noninterpretive ones (i.e., reference intake) [31]. Other reviews suggested FOPL can reduce sugar consumption [32, 33]. However, evidence assessing equity impact of FOPL remains limited. One systematic review examined the impact of FOPL on consumer understanding and usage across SES but did not examine the equity impacts of FOPL on actual sugar consumption and purchase behavior [34]. This systematic review aims to address this gap by summarizing and evaluating the impact of FOPL on social inequality in sugar consumption and purchase.

## 2 Methods

This systematic review followed the PRISMA (Preferred Reporting Items for Systematic Reviews and Meta‐Analyses) 2020 Checklist [35] (See Table S1 for PRISMA checklist) and was prospectively registered in the PROSPERO database (registration number: CRD42024526208) [36].

### 2.1 Data Sources and Search Strategy

Four electronic databases were searched including Ovid MEDLINE, Scopus, EMBASE, and Web of Science. The search strategy contained terms related to the FOPL (e.g., WL, keyhole label, and HSR) and high‐sugar containing foods and beverages such as chocolate, cookies, and SSBs (see Tables S2.1–S2.4 for full search strategy). Terms related to SES were not included to avoid unintentionally excluding studies that may have reported outcomes based on SES within their subgroup analysis. The last search was run on June 26, 2024.

### 2.2 Eligibility Criteria

Studies were eligible if they examined the impact of FOPL on social inequality in sugar consumption and purchase and were published in the English language since 2005. The population of interest was the general population, excluding studies focused on clinical groups, individuals with specific medical conditions, or those in institutional settings. The intervention included any types of FOPL implemented in observational settings or experimental environments that simulate real‐world shopping conditions. In the experimental setting, participants were expected to give cash or cards to make purchases. Studies assessing the impact of additional interventions, such as health promotion on sugar consumption, are excluded as their effect on sugar consumption may not be solely attributable to FOPL. To be eligible, studies needed a comparison group of participants who were unexposed to an FOPL intervention. Studies that only compared different types of FOPL without an unexposed control group were excluded. The outcome of interest was the difference in actual sugar consumption across SES subgroups, measured through purchasing behavior (e.g., sales data), and consumption behavior (e.g., sugar intake). SES was defined using individual level (e.g., income, education, and occupation) or area level (e.g., Socio‐Economic Indexes for Area) measures [37]. Studies that did not report sugar consumption or purchase by SES but instead focused solely on other health‐related outcomes—such as food choice, purchase intention, risk perception, or labeling comprehension—were excluded. Eligible study designs included randomized controlled trials (RCTs), observational studies (cross‐sectional, cohort, natural experiments, ecological studies), and simulation studies. Reviews, qualitative studies, and conference abstracts, gray literature, and so on were excluded. More details can be found in Table S3.

### 2.3 Study Selection

Search results from each database were exported to Covidence for removing duplicates and screening [38]. At least two reviewers independently screened the titles and abstracts, and full text of the articles to determine eligibility for inclusion. Any conflicts between reviewers were initially discussed, with additional unresolved conflicts adjudicated by a third reviewer A.S.

### 2.4 Data Extraction

The data extraction form was developed based on relevant literature and refined through discussion with the research team. To test clarity and consistency of the form, 20% of the studies was piloted. Z.G. extracted relevant data using Excel, and G.K. independently cross‐checked all the extracted data for accuracy. The extracted information included bibliographic data, study design, population characteristics, intervention specifics (e.g., any format of FOPL implemented and duration of exposure), details on the control group (e.g., no label and alternative interventions), SES measurement (e.g., income, education, or area‐level deprivation), data collection methods (survey or sales data), key outcomes (purchasing behavior or sugar intake), study findings, and conclusion related to the impact of FOPL on social inequality in sugar purchase and consumption.

### 2.5 Risk of Bias Assessment

The risk of bias in included studies was assessed using appropriate tools based on their study design. The risk of bias in non‐randomized studies—of exposures (ROBINS‐E tool) was utilized to assess the risk of bias in observational studies where the effect of an exposure on an outcome was investigated [39]. ROBINS‐E evaluates potential risk of bias across seven domains: (1) confounding factors; (2) measurement of exposure; (3) selection of participants into the study; (4) post‐exposure interventions; (5) missing data; (6) measurement of the outcome; and (7) selection of reported results. For RCTs, a risk‐of‐bias tool for randomized trials (RoB2) was employed [40]. RoB2 is structured into five domains: (1) randomization process; (2) deviation from intended interventions; (3) missing outcome data; (4) measurement of the outcome; and (5) selection of reported results. Both tools use signaling questions within each domain of bias, with response options: (1) yes; (2) probably yes; (3) probably no; (4) no; (5) no information. The signaling questions map responses onto a proposed risk‐of‐bias judgments for each domain. For ROBINS‐E, judgments include low risk of bias, some concerns, high risk of bias, and very high risk of bias. For RoB2, the possible risk‐of‐bias judgments are low risk of bias, some concerns, and high risk of bias.

The Consolidated Health Economic Evaluation Reporting Standards 2022 (CHEERS 2022) was used to check the transparency and consistency of included simulation studies [41]. CHEERS 2022 comprises 28 items across seven main domains: (1) title; (2) abstract; (3) introduction; (4) methods; (5) results; (6) discussion and (7) other relevant information. As CHEERS 2022 does not use a scoring system, we conducted a qualitative assessment of reporting completeness for health economics evaluations or simulation models using decision analysis (Table S4). The risk‐of‐bias assessments were performed independently by Z.G. and cross‐checked by G.K.

### 2.6 Data Synthesis

We provided a narrative synthesis of the results and grouped them according to the type of FOPL the studies evaluated. Due to the high risk of bias and high heterogeneity in outcomes such as sugar consumption and purchase measurement reported in included studies, a meta‐analysis was not conducted.

### 2.7 Declaration of Artificial Intelligence Generated Content (AIGC) Tools Usage

During the preparation of the manuscript, authors used GPT‐4 to check the grammar. No AIGC tools were used for developing the content. The authors critically reviewed and edited all content to ensure its accuracy and alignment with the evidence.

## 3 Results

### 3.1 Search Results

A total of 15,269 unique studies were identified by the systematic search across four databases (Figure 1). After removing duplication (*n* = 7568), 7701 studies were screened for eligibility. Of these, 7551 irrelevant papers were excluded after screening the titles and abstracts. The full text of 150 studies was reviewed, with 10 studies meeting the inclusion criteria. The most frequent reasons for exclusion were (1) the absence of reported results on social inequality in sugar consumption or purchase and (2) no real sugar consumption or purchase.

**FIGURE 1:**
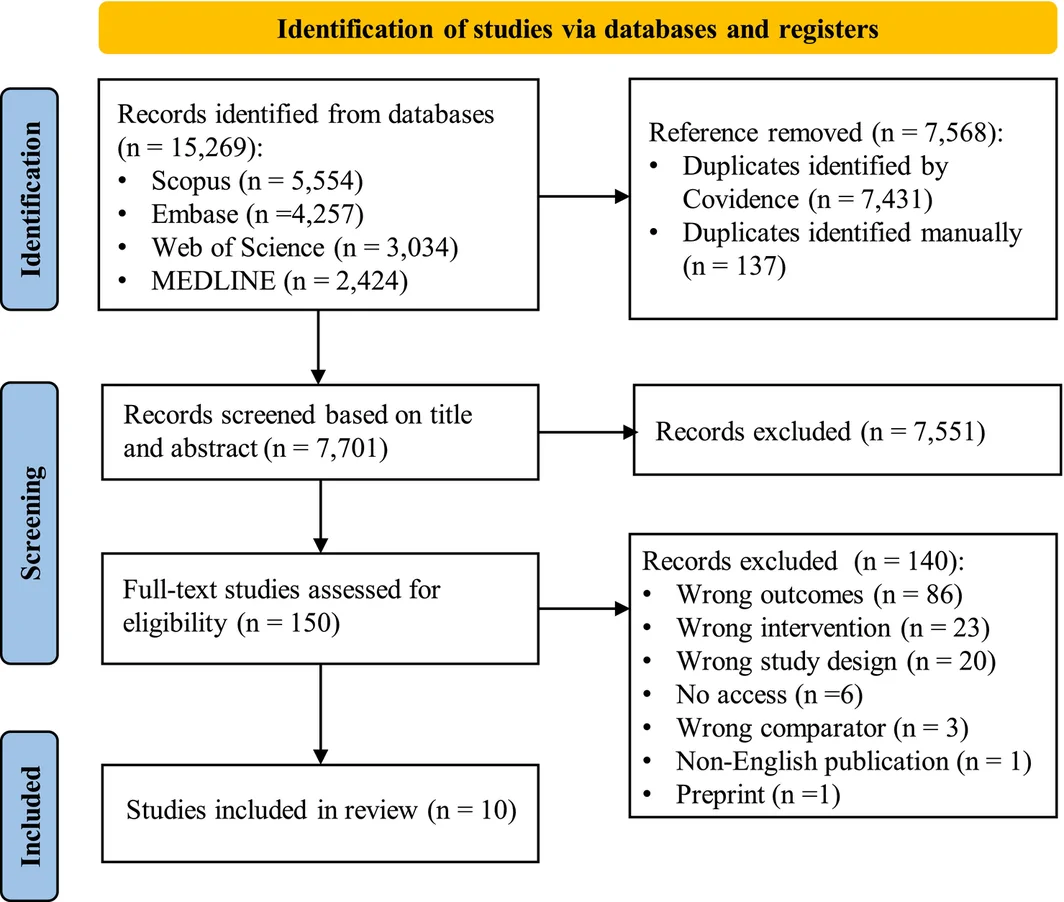
PRISMA study flow chart.

### 3.2 Characteristics of Included Studies

We included 10 studies in this review [42, 43, 44, 45, 46, 47, 48, 49, 50, 51]. A description of study characteristics is shown in Table 1. All included studies were conducted in high‐ and upper‐middle‐income countries. A total of four studies were conducted in the US [42, 43, 44, 45], four in Chile [46, 47, 48, 51], and one each in Sweden [49], and Ecuador [50]. The included studies were predominantly observational (*n* = 7) [42, 46, 47, 48, 49, 50, 51], followed by three RCTs [43, 44, 45]. The sample size varied across studies, ranging from 326 participants to 2383 households. Five studies reported the age distribution of participants. Out of these, four studies primarily focused on adults [42, 43, 44, 45], and one only included students at Years 5, 8, and 11 [49]. Five studies reported the age and gender distribution of the sample [42, 43, 44, 45, 49]. The majority of participants in those studies were females.

**TABLE 1:** Characteristics of included studies.

First author, year	Study design	Country	Sample size (*n*) male (%) female (%)	Ethnicity and race (%)	Age (years)	Type of FOPL	Measurement of SES	Sugar consumption and purchase measurement	Data collection of sugar consumption and purchase
Grummon 2019 [43]	RCT	US	400 participants 39.75% 59.0%	White (45%) Black or African American (22.25%) Asian (24.5%) Other/multiracial (7.25%)	Mean: 29.0 (10.3)	WL	Income, education, and race	SSB calories purchased	Purchase data in experimental setting
Grummon 2019 [42]	Simulation study	US	NR	Non‐Hispanic White (NR) Non‐Hispanic Black (NR) Hispanic/Mexican American (NR)	Range: 18–39 and 40–65	SSB WL	Education and income	SSB intake	Simulation‐generated data
Hall 2022 [44]	RCT	US	326 parents 23% 77%	Non‐Hispanic White (control: 46%; intervention: 44%) Non‐Hispanic Non‐White (control: 35%; intervention: 34%) Hispanic (control: 20%; intervention: 21%)	Mean: 38	Pictorial warnings	Parental educational attainment, parental annual household income	Sugary drinks selection	Point‐of‐sale system record
Musicus 2022 [45]	RCT	US	5005 participants 28.3% 71.7%	Asian (control: 3.5%; intervention: 3.7%) Black or African American (control: 21%; intervention: 23.5%) Hispanic or Latinx/o/a (control: 22.4%; intervention: 21.4%) Native American (control: 1.0%; intervention: 1.7%) Native Hawaiian, Pacific Islander, or Alaska Native (control: 1.4%; intervention: 0.6%) White (control: 50.8%; intervention: 50.3%) Other (control: 6.4%; intervention: 6.0%)	Mean: 31.5 (8.3)	WL	WIC participants	Selection of a high‐added sugar beverage elected beverages' calories and added sugar (in grams) content	Experimental measurement
Paraje 2023 [51]	Before and after study	Chile	The average number of total households (monthly) was 1933.45 NR NR	NR	NR	WL	Socioeconomic status (from high to low: ABC1, C2C3 and DE)	Volume purchased beverages with high in sugar label	Purchases data
Rebolledo 2022 [46]	Before and after study	Chile	2380 individual households NR NR	NR	NR	WL	Education, household assets	NNS sweetness, total sugars sweetness, and total sweetness, all in terms of sucrose equivalent/capita/day.	Purchased data collected by through barcode scanning, manual item logging, receipts review, household pantry inventories, and inspections empty product packages
Sandoval 2019 [50]	Before and after study	Ecuador	1646 households NR NR	NR	NR	TLS	Socioeconomic status (high and medium)	Mean expenditures of carbonated soft drinks	Purchases data
Taillie 2021 [47]	Before and after study	Chile	2009 households NR NR	NR	NR	WL	Education, household assets	Overall mean total calories purchased from high‐in food and beverages	Sales data
Taillie 2020 [48]	Before and after study	Chile	2383 households NR NR	NR	NR	WL	Head‐of‐household education, tertile of household assets index	Purchase high in beverage	Sales data
Wansellus 2022 [49]	Simulation study	Sweden	3099 participants 45% 55%	NR	Age range: school years 5, 8 and 11	Keyhole	Highest attained parental education level (≤ 12 years, > 12 years, and missing)	Free sugars	Self‐reported for business as real scenario

Abbreviations: FOPL, front‐of‐pack labeling; GDAs, guideline daily amounts; NR, not reported; RCT, randomized controlled trials; SES, socioeconomic status; TLS, traffic light system; US, United States of America; WIC, Special Supplemental Nutrition Program for Women, Infants, and Children; WL, warning label.

With regard to the type of FOPL examined, WL was the most common (*n* = 8) [42, 43, 44, 45, 46, 47, 48, 51], followed by Keyhole label (*n* = 1) [49], and Traffic Light System (*n* = 1) [50]. The exposure window for WL ranged from the time of single exposure to 30 months, whereas it was 16 months for the Traffic Light System.

There was high heterogeneity in the measurement of sugar consumption and purchase across studies. Most studies measured beverage‐specific sugar consumption or related expenditures, such as SSB calories purchased, SSB intake, and sugary drinks [42, 43, 44, 45, 48, 50, 51]. Three studies measured overall sugar consumption [46, 47, 49]. Specifically, overall sugar consumption and purchase were assessed using total calories from high‐in food and beverage purchases [47], total free sugar intake [49], and combined purchase of non‐nutritive sweetener sweetness and total sugar sweetness [46]. The data on sugar purchase and consumption were collected by using purchase data [46, 50, 51], sales records [44, 47, 48], self‐reported data [49], experimental records [43, 45], and simulation data [42]. To assess FOPL's impact on social inequality in sugar consumption, seven studies stratified their analysis by SES [42, 43, 44, 45, 49, 50, 51], two examined the joint effect of SES and FOPL [46, 47], and one employed both approaches [48]. SES was measured by single indicators such as income [42, 43, 44, 46, 50, 51], education [42, 43, 44, 46, 47, 48, 49], assets [46, 47, 48], ethnicity/race [42, 43, 45], and a composite indicator—SES [50, 51].

### 3.3 Types of Front‐of‐Pack Labeling

#### 3.3.1 Warning Label

Eight studies reported that WLs reduce high sugar consumption and purchase across whole populations in the US and Chile (Table 2) [42, 43, 44, 45, 46, 47, 48, 51]. Seven out of eight papers investigated the stratified effect of WL on sugar consumption by socioeconomic subgroups without reporting baseline sugar consumption and social inequality in sugar consumption. The impact of WL on social inequality in sugar consumption is inconsistent.

**TABLE 2:** The impact of FOPL on social inequality in sugar consumption.

First author, year	Method	Details of FOPL	Effect estimate	Findings (methodological approach)	Conclusion on social inequality
Warning label (nutrient‐specific systems and interpretive)					
Grummon 2019 [43]	Two‐part model	Health warning labels displayed the message “WARNING: Beverages with added sugar contribute to tooth decay, diabetes, and obesity” in white text on a red octagon (1.5‐inch‐wide span) with a thin white border	Predicted mean difference	Income (stratification): $0–$24,999: −40.9 cal/day; $25,000–$49,999: −25.4 cal/day; $50,000–$74,999: 19.9 cal/day; ≥$75,000: −47.9 cal/day	U‐shaped effect
				Education (stratification): College educational attainment or higher: −26.7 cal/day; Some college or less educational attainment: −58.2 cal/day	Stronger effect on education‐ disadvantaged group
				Ethnicity (stratification): Non‐Hispanic: −30.6 cal/day; Hispanic: −46.7 cal/day	Stronger effect on Hispanic subgroup
				Race (stratification): White: −36.0 cal/day; Black or African American: −34.2 cal/day; Asian: −25.8 cal/day; Other or multiracial: −5.1 cal/day	Stronger effect on White subgroup followed by Black or African American subgroup
Grummon 2019 [42]	A stochastic microsimulation model	SSB health warning label	Estimated changes and absolute difference	Income (stratification): High income: −22.3 cal/day; Low income: −32.9 cal/day Difference between Household income ≤ 185% and > 185% of Federal Poverty Level: 10.6 (95% UI: 7.4, 13.9) kcal/day	Stronger effect on income‐disadvantaged group
			Estimated changes and absolute difference	Education (stratification): Low educational attainment: −30.4 cal/day; High educational attainment: −16.3 cal/day; Difference between Education of ≤ some college and ≥ college degree: 14.1 (95% UI:10.9, 17.4) kcal/day	Stronger effect on education‐disadvantaged group
			Estimated changes	Ethnicity (stratification): White: −23.6 cal/day; Black: −30.4 cal/day; Hispanic: −27.9 cal/day	Stronger effect on Black subgroup, followed by Hispanic subgroup
Hall 2022 [44]	Logistic regression models	Approximately half of the sugary drinks displayed the heart damage warning label, and the other half displayed the type 2 diabetes label.	Percent of parents selecting sugary drinks	Parental educational attainment (stratification): Intervention: 30% for < 4 year college degree; 27% for ≥ 4 year college degree; Control: 47% for < 4 year college degree; 45% for ≥ 4 year college degree	No difference in effect based on parental educational attainment levels
			Percent of parents selecting sugary drinks	Race/ethnicity (stratification): Intervention: 26% for Non‐Hispanic White, 34% for Non‐Hispanic Non‐White, and 24% for Hispanic Control: 42% for Non‐Hispanic White, 56% for Non‐Hispanic Non‐White, and 29% for Hispanic	Stronger effect on Non‐Hispanic and Non‐White subgroup
Musicus 2022 [45]	Linear regression models	High‐added sugar warning, teaspoons of added sugar disclosure	Interaction	Race/ethnicity (stratification): Hispanic participants selected beverages with fewer calories than non‐Hispanic participants in the warning (*β* = −13.1; *p* for interaction = 0.02) conditions compared with participants in the control group.	Stronger effect on Hispanic subgroup
Rebolledo 2022 [46]	Longitudinal fixed‐effects models	Warning labels with the words “high in” sugar, sodium, saturated fat, and/or calories	Sweetness changes	Household assets (interaction): Absolute difference of nonnutritive sweetener sweetness purchases (sucrose equivalents) between counterfactual post‐policy and post‐policy across: Low household assets: 2.4; Middle household assets: 2.6; High household assets: 3.1. Absolute difference of total sweetener sweetness purchases (sucrose equivalents) between counterfactual post‐policy and post‐policy: Low household assets: −2.7 Middle household assets: −2.2 High household assets: −2.3 Absolute difference of total sweetness purchases (sucrose equivalents) between counterfactual post‐policy and post‐policy: Low household assets: −0.3 Middle household assets: 0.4 High household assets: 0.8	Stronger effect on the household asset‐disadvantaged group
			Sweetness changes	Education (interaction): Absolute difference of nonnutritive sweetener sweetness (sucrose equivalents) purchases between counterfactual post‐policy and post‐policy: < High school education: 2.8; high school education:2.4; ≥ college:3.1 Absolute difference of total sweetener sweetness (sucrose equivalents) purchases between counterfactual post‐policy and post‐policy: < High school education: −2.2; high school education: −2.0; ≥ college:−3.3 Absolute difference of total sweetness (sucrose equivalents) purchases between counterfactual post‐policy and post‐policy < high school education: 0.6; high school education: 0.4; ≥ college: −0.2	Stronger effect on education‐ advantaged group
Taillie 2021 [47]	Fixed‐effects linear regression model	Warning labels consisted of black octagons with the words high‐in sugar, sodium, saturated fat, and/or calories, whichever applies	Mean total high‐in food and beverage calories purchased	Education (interaction): Mean total calories from high‐in food and beverage purchases: > High school education: 208.1 (95% CI: 198.1, 218.1) before the FOPL intervention and 156.1 (95% CI: 148.0, 164.2) after the intervention. High school education: 208.3 (95% CI: 200.6, 216.0) before the FOPL intervention and 154.6 (95% CI: 148.4, 160.8) after the intervention. ≥ college: 233.0 (95% CI: 223.2, 242.9) before the FOPL intervention and 164.1 (95% CI: 157.4, 170.8) after the intervention	Stronger effect on education‐advantaged group
			Mean total high‐in food and beverage calories purchased	Household assets (interaction): Mean total calories from high‐in food and beverage purchases: Households with low household assets index: 210.1 (95% CI: 201.2, 219.1) before the FOPL intervention and 155.6 (95% CI: 148.9, 162.3) after the intervention; Middle household assets index: 209.2 (95% CI: 200.8, 217.6) before the FOPL intervention and 157.1 (95% CI: 150.5, 163.6) after the intervention; High household assets index was 228.9 (95% CI: 219.8, 238.1) before the FOPL intervention and 162.1 (95% CI: 155.1, 169.1) after the intervention.	Stronger effect on household assets‐advantaged group
Taillie 2020 [48]	Fixed‐effect model and F‐test	Warning labels consist of a black octagon with white borders placed on the front of the food or beverage package, including the words “alto en…” (“high in…”) calories, sugars, saturated fats, or sodium	Absolute and relative changes in purchases of high‐in beverages	Head‐of‐household education (stratification): Reduction of high‐in beverages purchases: < High school degree: 27.2 ml/day per capita (21.5%); high school degree: 20.9 mL/day per capita (19.5%); ≥ college degree: 27.7 mL/day per capita (28.7%)	Stronger effect on education‐advantaged group
			Interaction	Head‐of‐household education (interaction): The coefficient of post‐regulation × month/year/completed high school was 0.0004 [95% CI: −0.01, 0.002] and of post‐regulation × month/year/college or greater was −0.003 [95% CI: −0.005, −0.001].	Stronger effect on head‐of‐household education‐advantaged group
Paraje 2023 [51]	A household fixed effect before–after model	NR	Marginal effect of the intervention on the volume of beverages before and after the intervention	SES (from high to low: ABC1, C2C3 and DE) (stratification): The volume of beverages purchased decreased by 4543.91 mL in the ABC1 group, 3720.71 mL in the C2C3 group, and 2846.33 mL in the DE group after warning label intervention.	Stronger effect on SES‐advantaged group
Traffic Light System (nutrient‐specific system and interpretive indictor)					
Sandoval 2019 [50]	Nonlinear almost ideal demand system	Color‐code TLS consisted of the content of sugar, fat, and salt	Effects of the demand shifters on mean expenditures ($ per‐capita per month)	SES (high and medium) (stratification): The quantity purchased of Coca‐Cola (reference group: low and very low SES): High SES: −0.132 L per‐capita per month; medium SES: 0.022 L per‐capita The quantity purchased of dark colored high‐sugar beverage (reference group: low and very low SES): High SES: −0.193 L per‐capita per month; medium SES: 0.135 L per‐capita per month The quantity purchased of low‐and non‐sugar (reference group: Low and very low SES): High SES: 0.035 L per‐capita per month; medium SES: 0.013 L per‐capita per month The quantity purchased of all other high sugar sodas (reference group: low and very low SES): High SES: −0.151 L per‐capita per month; medium SES: 0.020 L per‐capita per month	U‐shaped effect
Keyhole (summary and noninterpretive indicator)					
Wansellus 2022 [49]	Logistic regression analysis	NR	Group difference in free sugar	Parental education (stratification): No differences were observed regarding intakes of free sugars.	No difference in effect based on parental educational attainment levels

Abbreviations: FOPL, front‐of‐pack labeling; NR, not reported; SES, socioeconomic status; SSB, sugar‐sweetened beverage; TLS, traffic light system; UI, uncertainty interval.

WL formats varied across studies conducted in the US. Two studies evaluated labels that included both high‐sugar warnings and NCD risk information [43, 44], one study used labels with sugar content warnings only [45], and one study did not report the label format [42]. Grummon et al. observed a U‐shaped association between income and the predicted difference in SSB purchases following WL implementation, with the greatest reductions among the lowest (< $25,000: −40.9 kcal/day) and highest (≥ $75,000: −47.9 kcal/day) income groups. Smaller reductions were observed in the $25,000–$49,999 group (−25.4 kcal/day), while an increase in the $50,000–$74,999 group (+19.9 kcal/day) [43]. Reduction in SSB purchases was also more pronounced among participants with some college education or less who reduced their SSB purchases by 58.2 kcal/day, compared with a reduction of 26.7 kcal/day among those with college education or higher. A simulation study further predicted that a national WL policy would lead to a greater reduction in SSB consumption among low‐income participants over 5 years in the US [42]. Participants with lower educational attainment were predicted to reduce their SSB intake by 14.1 kcal/day more than college graduates. Hall et al.'s findings aligned with these results, showing that individuals with lower education levels were more responsive to WLs [44].

Four studies conducted in the US also examined the impact of WLs on social inequality through a race/ethnicity perspective. All studies reported that WLs led to a reduction in sugar consumption and purchase across all racial/ethnic groups, though the findings of inequality were not entirely consistent. Hall et al. observed the largest reduction in sugary drink selection among Non‐Hispanic White parents (from 42% to 26%) and the smallest reduction in Hispanic parents (from 29% to 24%) exposed to WLs compared with the control group [44]. This finding is consistent with Grummon et al., where the largest reduction in SSB purchases was observed in White participants (−36 kcal/day) between the WL and control group compared with Black or African American, Asian, and other/multiracial subgroups [43]. However, the finding from a simulation study indicated that Black participants decreased more SSB consumption (−30.4 kcal/day) compared with White (−23.6 kcal/day) and Hispanic participants (−27.9 kcal/day) over 5 years [42]. Musicus et al. found that Hispanic participants consumed fewer calories of beverages when exposed to the WL compared with the control [52].

The WLs in Chile uniformly employed black octagons with the wording “high‐in sugar” [46, 47, 48], with one paper omitting label design details [51]. Interestingly, three out of four studies conducted in Chile found a positive association between SES and sugar consumption at baseline, where more educated and affluent subgroups consumed more sugar [47, 48, 51], with one without baseline sugar information [46]. All studies in Chile used secondary data from Kantar Worldpanel which are longitudinal data on household food purchases. Three studies analyzed 18 months of data before and after the implementation of health warning labels [46, 47, 48]. One study covered 29 months before and 19 months after the policy [51]. Fixed effects analysis showed overall reductions in trends of high sugar consumption after policy implementation. However, the stratified analysis showed that the effect was more pronounced in affluent subgroups. Eighteen months of exposure to WLs led to a decrease of 27.2 mL per capita per day for households with less than a high school degree, 20.9 mL for those with a high school degree, and 27.7 mL for those with a college degree or higher compared with counterfactuals [48]. Similarly, households with high household assets had the largest calorie reduction (66.8 kcal), followed by low asset (54.5 kcal), and middle asset households (52.1 kcal). The estimated absolute difference of total sweetener sweetness (sucrose equivalents) purchases between counterfactual post‐policy and post‐policy across education categories (less than high school, high school, and college or greater) were −2.2, −2.0, and −3.3 [46]. Across household asset categories, the estimated absolute difference was −2.7 for low‐asset households, −2.2 for middle‐asset households, and −2.3 for high‐asset households. After 19 months of implementation of WLs in Chile, people from the high SES group reduced their beverage consumption by 1697.58 mL more than those from a low SES [51]. Households where head of household had college or greater education had the highest calorie reduction from high‐in food and beverage purchases (68.9 kcal), followed by those with high school education (53.7 kcal) and those with less than high school education (52 kcal) [47].

#### 3.3.2 Other Types of FOPLs

Two studies used other types of FOPLs and showed variable effects on social inequality. Wansellus et al. reported no significant differences in free sugar intake across SES groups following the implementation of Keyhole labels, suggesting that Keyhole labels had a uniform effect regardless of SES [49]. In contrast, Sandoval et al. observed high SES households exhibited a greater reduction in high‐sugar beverage purchases—such as Coca‐Cola (−0.132 L/month), dark‐colored high‐sugar drinks (−0.193 L/month), and other high‐sugar sodas (−0.151 L/month) [50]. Medium SES households showed increases in the purchase of some high‐sugar beverages (e.g., +0.135 L/month for dark‐colored high‐sugar beverages), suggesting a U‐shaped association between SES and purchasing changes. These findings suggest that the impact of TLS may be uneven across SES groups.

### 3.4 Risk of Bias

Five papers were assessed for risk of bias using the ROBINS‐E tool [46, 47, 48, 50, 51], three papers using the ROB2 tool (Table 3 and Table 4) [43, 44, 45], and two employing CHEERS [42, 49].

**TABLE 3:** Risk of bias for observational studies using ROBINS‐E.

First author and year of publication	Risk of bias due to confounding	Risk of bias arising from measurement of the exposure	Risk of bias in selection of participants into the study	Risk of bias due to post‐exposure interventions	Risk of bias due to missing data	Risk of bias arising from measurement of the outcome	Risk of bias in selection of the reported result	Overall
Rebolledo 2022 [46]	High	Low	Low	Low	Low	High	Low	Very high
Taillie 2021 [47]	Low	Low	Low	Low	Low	Low	Low	Low
Taillie 2020 [48]	Low	Low	Low	Low	High	Some concerns	Low	High
Sandoval 2019 [50]	High	Low	Low	Low	High	Low	Low	Very high
Paraje 2023 [51]	High	Low	Low	Low	Low	Low	Low	High

**TABLE 4:** Risk of bias for randomized control trials studies using ROB2.

First author and year of publication	Risk of bias arising from the randomization process	Risk of bias due to deviations from the intended interventions	Missing outcome data	Risk of bias in measurement of the outcome	Risk of bias in selection of the reported result	Overall
Grummon 2019 [43]	Low	Low	Low	Low	Low	Low
Hall 2022 [44]	High	Low	Low	High	Low	High
Musicus 2022 [45]	Low	High	Low	Low	Low	High

Of the five studies assessed by the ROBINS‐E tool, two studies were classified as having a very high risk of bias [46, 50], two studies as having a high risk of bias [48, 51], and one study as having a low risk of bias [47]. There was consistent evidence of high bias due to non‐adjustment of confounding factors, as most studies failed to control for important variables such as age and sex, which we considered a minimum set of confounders. All five studies were rated as low risk of bias in the domains of exposure measurement, participant selection, post‐exposure interventions, handling of missing data, outcome measurement, and reporting of results. However, two studies were identified as having a high risk of bias due to missing data [48, 50]. Neither study provided information on how missing data were handled or accounted for. Rebolledo et al.'s study showed a high risk of bias in outcome measurement, as it relied on scanning receipts and store packages to assess sugar consumption, a method that may introduce bias if, for example, households with higher SES are less likely to retain receipts [46]. Finally, Sandoval et al.'s study raised concerns regarding the measurement of outcomes because no details were provided on how outcomes were measured, leading to uncertainty about the risk of bias in this domain [50].

Two out of three RCTs were classified as having high risk of bias [44, 45], and one as low risk of bias [43]. The sources of high risk of bias varied across RCTs. Hall et al.'s study exhibited a high risk of bias due to issues with the randomization process and outcome measurement [44]. Musicus et al.'s study had a high risk of bias due to deviations from the intended interventions [45].

For the two simulation studies, Grummon et al.'s study provided a clear description of background and discussion [42]. However, the study did not report perspective, discount rate, measurement, and valuation of resources and costs, currency, price date and conversion rate, and approach to and effect of engagement with stakeholders affected by the study. Wanseliu et al.'s paper provided limited information, with several key checklist items not being reported [49].

## 4 Discussion

This systematic review examined the impact of FOPL on social inequality in sugar consumption. All studies reported that FOPL reduced sugar consumption at the population level, yet the findings of their impact on social inequality in sugar consumption yielded inconsistent evidence. The results showed mixed patterns of the impact on SES, with some studies demonstrating more beneficial effects in socially advantaged groups, others in socially disadvantaged groups, some reporting U‐shaped effects, and some reporting no significant differences between subgroups. The overall quality of evidence was limited by a high risk of bias in most studies, underscoring the need for higher‐quality studies to quantify FOPL's effect on socioeconomic inequalities in sugar consumption.

Four reasons may explain the inconsistent findings regarding the impact of FOPL on social inequality in sugar consumption and purchase. First, variations in the intervention period may influence FOPL's differential effects across SES strata. For instance, Grummon et al. conducted an RCT in which participants were exposed to FOPL only once, finding greater reductions in sugar consumption among individuals experiencing high SES compared with low SES [43]. However, their simulation model suggested that participants experiencing low SES would experience larger reductions in caloric intake from sugar over 5 years [42]. Second, 60% of the included studies failed to report baseline sugar consumption, social inequalities in baseline sugar consumption or both, limiting the ability to assess how socioeconomic inequality in consumption changed over time and potentially introducing bias. Third, some studies included non‐representative samples. For example, Tallie et al. reported an overrepresentation of high‐SES participants in their sample [47, 48], which potentially impacts the external validity of their results. Last, there is limited evidence on how sugar consumption and purchasing patterns change over time across different SES groups following the introduction of FOPL.

Our systematic review found that FOPL can reduce sugar consumption and purchase at the population level. This finding is consistent with previous studies. Croker et al.'s meta‐analysis of experimental studies showed that FOPL significantly reduced the sugar content of purchases compared with the absence of FOPL (−0.40 g/100 g, *p* < 0.01) [32]. Gupta et al. found that graphic and text warning labels significantly decreased both intended and actual purchase of SSB [33]. Shrestha et al. conducted a narrative review that included two papers that were stratified analyses of the effectiveness of FOPL on actual consumption and purchases across SES, one of which is also included in our review. We excluded the other study because it assessed the impact of FOPL on the healthiness of food purchases rather than sugar consumption and purchase. However, they did not specifically focus on sugar consumption or purchase. Their findings suggest that individuals with lower SES are less likely to benefit from FOPL in terms of actual healthy purchase. These findings highlight the importance of examining equity impact of FOPL. While FOPL provides clear benefits at the population level, it is crucial to understand how different SES groups respond to it. Incorporating a social equity perspective is essential not only for enhancing the public health impact of FOPL but also for ensuring that this strategy contributes meaningfully to health justice.

Future research should examine the impact of different types of FOPL on social inequality in sugar consumption across different settings, especially in low‐income countries. High‐quality quantitative studies, for example, longitudinal studies with baseline social inequality in sugar consumption and purchase, are needed to understand whether FOPL reduces social inequality in sugar consumption and purchase behavior. To better inform policymakers and support the implementation of FOPL policies, further research is also required to examine how changes in sugar consumption and purchase across SES groups affect the broader burden of sugar‐related health outcomes and their associated inequality. Lastly, more transparent simulation studies involving more stakeholder perspectives are needed to inform the long‐term impact of FOPL on social inequality in sugar consumption and purchase.

Overall, FOPL should be prioritized in sugar reduction and obesity strategies, given its effectiveness in reducing sugar consumption and purchase at the population level. However, evidence of its impact in addressing social inequality in sugar consumption remains limited and inconsistent.

This is the first review to systematically examine the impact of FOPL on social inequality in sugar consumption, thereby addressing a significant research gap. We specifically looked at social inequality in actual sugar consumption and purchases, which can capture the impact of FOPL on real behavior change. However, this systematic review also has limitations. Only English publications were included. This may result in language bias and the exclusion of potentially relevant studies published in other languages. Due to the overall high risk of bias and high heterogeneity between the included studies, we were unable to conduct a meta‐analysis.

There are some further limitations of the included studies. Most studies identified in our review were from high‐ and upper‐middle‐income countries, which limits the generalizability of our findings to low‐income countries. Also, most included studies examined the impact of WLs on social inequality in sugar consumption, while the effects of other FOPLs, such as Nutri Score and Healthy Choice logos, remain unclear. Moreover, the included studies primarily examined the impact of FOPL on social inequality in sugar consumption and purchase by effect modification instead of joint effect or mediation analysis, which limits the ability to conclude whether and how FOPLs interact with SES to reduce inequality in sugar consumption or purchasing behaviors. The data used in the included studies are limited by the lack of long‐term, individual‐level information on sugar purchase and consumption. Individual level data are essential for identifying a more precise association between FOPL and individual sugar purchase and consumption across SES subgroups over time.

## 5 Conclusion

Our review found that FOPL can reduce sugar consumption at the population level. However, its effects on social inequality in sugar consumption remain unclear due to inconsistent findings and limitations in the existing evidence. More high‐quality research is needed to understand how different types of FOPL affect sugar consumption across socioeconomic groups, particularly in low‐income countries.

## Funding

This work was supported by China Scholarship Council–University of Melbourne PhD scholarship (202308240005).

## Conflicts of Interest

The authors declare no conflicts of interest.

## Supporting information


**Table S1:** PRISMA writing checklist.
**Table S2:1.** Search strategy used in Ovid Medline.
**Table S2:2.** Search strategy used in Web of Science.
**Table S2:3.** Search strategy used in Scopus.
**Table S2:4.** Search strategy used in EMBASE.
**Table S3:** Eligible criteria.
**Table S4:1.** Assessment of Reporting Quality Using the CHEERS 2020 Checklist: Grummon et al. 2019.
**Table S4:2.** Assessment of Reporting Quality Using the CHEERS 2020 Checklist: Wanseliu et al. 2022.

## Data Availability

Data sharing not applicable to this article as no datasets were generated or analysed during the current study.
