# Supplementary material for: The Impact of Front‐of‐Pack Labeling on Social Inequality in Sugar Consumption and Purchase: A Systematic Review
Source: Obes Rev. 2026 May 19;27(11):e70150. doi: 10.1111/obr.70150 (PMC13655994; doi:10.1111/obr.70150)
Supplement: Supplementary file 1 [file OBR-27-e70150-s001.docx]

**Title**: The impact of front-of-pack labelling on social inequality in sugar consumption and purchase: A systematic review

**Authors:** Ziyao Ge^1,*^, Claudia H. Marck^2^, Adyya Gupta^3^, Gagandeep Kaur^1,4,5^, Roisin McGrath^6^, Ankur Singh^4,5^

**Affiliations:** ^1^Centre for Epidemiology and Biostatistics, Melbourne School of Population and Global Health, The University of Melbourne, Victoria, Australia; ^2^Disability and Health Unit, Centre for Health Policy, Melbourne School of Population and Global Health, The University of Melbourne, Victoria, Australia; ^3^Deakin Centre for Global Preventive Health and Nutrition, Deakin Institute for Health Transformation, School of Health and Social Development, Faculty of Health, Deakin University, Victoria, Australia; ^4^University of Sydney School of Dentistry, The University of Sydney, New South Wales, Australia; ^5^Charles Perkins Centre, The University of Sydney, Sydney, New South Wales, Australia; ^6^Melbourne Dental School, The University of Melbourne, Victoria, Australia;

*Address for the corresponding authors:

Ziyao Ge, Centre for Epidemiology and Biostatistics, Melbourne School of Population and Global Health, The University of Melbourne, Victoria, Australia.

Email: ziyaog@student.unimelb.edu.au

**Supporting Information 1 – PRISMA writing checklist**

***Table S1. PRISMA writing checklist***

| **Section and Topic** | **Item #** | **Checklist item** | **Location where item is reported** |
| --- | --- | --- | --- |
| **TITLE** | | |  |
| Title | 1 | Identify the report as a systematic review. | Page 1 |
| **ABSTRACT** | | |  |
| Abstract | 2 | See the PRISMA 2020 for Abstracts checklist. | Page 1 |
| **INTRODUCTION** | | |  |
| Rationale | 3 | Describe the rationale for the review in the context of existing knowledge. | Page 1-2 |
| Objectives | 4 | Provide an explicit statement of the objective(s) or question(s) the review addresses. | Page 2 |
| **METHODS** | | |  |
| Eligibility criteria | 5 | Specify the inclusion and exclusion criteria for the review and how studies were grouped for the syntheses. | Page 2-3 |
| Information sources | 6 | Specify all databases, registers, websites, organisations, reference lists and other sources searched or consulted to identify studies. Specify the date when each source was last searched or consulted. | Page 2 |
| Search strategy | 7 | Present the full search strategies for all databases, registers and websites, including any filters and limits used. | Supporting information Table S2.1-2.4 |
| Selection process | 8 | Specify the methods used to decide whether a study met the inclusion criteria of the review, including how many reviewers screened each record and each report retrieved, whether they worked independently, and if applicable, details of automation tools used in the process. | Page 3 |
| Data collection process | 9 | Specify the methods used to collect data from reports, including how many reviewers collected data from each report, whether they worked independently, any processes for obtaining or confirming data from study investigators, and if applicable, details of automation tools used in the process. | Page 3 |
| Data items | 10a | List and define all outcomes for which data were sought. Specify whether all results that were compatible with each outcome domain in each study were sought (e.g. for all measures, time points, analyses), and if not, the methods used to decide which results to collect. | Page 3 |
|  | 10b | List and define all other variables for which data were sought (e.g. participant and intervention characteristics, funding sources). Describe any assumptions made about any missing or unclear information. | Page 3 |
| Study risk of bias assessment | 11 | Specify the methods used to assess risk of bias in the included studies, including details of the tool(s) used, how many reviewers assessed each study and whether they worked independently, and if applicable, details of automation tools used in the process. | Page 3 |
| Effect measures | 12 | Specify for each outcome the effect measure(s) (e.g. risk ratio, mean difference) used in the synthesis or presentation of results. | Page 3 |
| Synthesis methods | 13a | Describe the processes used to decide which studies were eligible for each synthesis (e.g. tabulating the study intervention characteristics and comparing against the planned groups for each synthesis (item #5)). | Page 3 |
|  | 13b | Describe any methods required to prepare the data for presentation or synthesis, such as handling of missing summary statistics, or data conversions. | N/A |
|  | 13c | Describe any methods used to tabulate or visually display results of individual studies and syntheses. | N/A |
|  | 13d | Describe any methods used to synthesize results and provide a rationale for the choice(s). If meta-analysis was performed, describe the model(s), method(s) to identify the presence and extent of statistical heterogeneity, and software package(s) used. | N/A |
|  | 13e | Describe any methods used to explore possible causes of heterogeneity among study results (e.g. subgroup analysis, meta-regression). | N/A |
|  | 13f | Describe any sensitivity analyses conducted to assess robustness of the synthesized results. | N/A |
| Reporting bias assessment | 14 | Describe any methods used to assess risk of bias due to missing results in a synthesis (arising from reporting biases). | N/A |
| Certainty assessment | 15 | Describe any methods used to assess certainty (or confidence) in the body of evidence for an outcome. | N/A |
| **RESULTS** | | |  |
| Study selection | 16a | Describe the results of the search and selection process, from the number of records identified in the search to the number of studies included in the review, ideally using a flow diagram. | Page 3, Figure 1 |
|  | 16b | Cite studies that might appear to meet the inclusion criteria, but which were excluded, and explain why they were excluded. | Page 3, Figure 1 |
| Study characteristics | 17 | Cite each included study and present its characteristics. | Page 3-4, Table 1 |
| Risk of bias in studies | 18 | Present assessments of risk of bias for each included study. | Page 13-14, Table 3 and Table 4 |
| Results of individual studies | 19 | For all outcomes, present, for each study: (a) summary statistics for each group (where appropriate) and (b) an effect estimate and its precision (e.g. confidence/credible interval), ideally using structured tables or plots. | Page 4-13, Table 2 |
| Results of syntheses | 20a | For each synthesis, briefly summarise the characteristics and risk of bias among contributing studies. | N/A |
|  | 20b | Present results of all statistical syntheses conducted. If meta-analysis was done, present for each the summary estimate and its precision (e.g. confidence/credible interval) and measures of statistical heterogeneity. If comparing groups, describe the direction of the effect. | N/A |
|  | 20c | Present results of all investigations of possible causes of heterogeneity among study results. | N/A |
|  | 20d | Present results of all sensitivity analyses conducted to assess the robustness of the synthesized results. | N/A |
| Reporting biases | 21 | Present assessments of risk of bias due to missing results (arising from reporting biases) for each synthesis assessed. | N/A |
| Certainty of evidence | 22 | Present assessments of certainty (or confidence) in the body of evidence for each outcome assessed. | N/A |
| **DISCUSSION** | | |  |
| Discussion | 23a | Provide a general interpretation of the results in the context of other evidence. | Page 14 |
|  | 23b | Discuss any limitations of the evidence included in the review. | Page 14 |
|  | 23c | Discuss any limitations of the review processes used. | Page 15 |
|  | 23d | Discuss implications of the results for practice, policy, and future research. | Page 14 |
| **OTHER INFORMATION** | | |  |
| Registration and protocol | 24a | Provide registration information for the review, including register name and registration number, or state that the review was not registered. | Page 2 |
|  | 24b | Indicate where the review protocol can be accessed, or state that a protocol was not prepared. | Page 2 |
|  | 24c | Describe and explain any amendments to information provided at registration or in the protocol. | N/A |
| Support | 25 | Describe sources of financial or non-financial support for the review, and the role of the funders or sponsors in the review. | Page 15 |
| Competing interests | 26 | Declare any competing interests of review authors. | Page 15 |
| Availability of data, code and other materials | 27 | Report which of the following are publicly available and where they can be found: template data collection forms; data extracted from included studies; data used for all analyses; analytic code; any other materials used in the review. | N/A |

*From:*  Page MJ, McKenzie JE, Bossuyt PM, Boutron I, Hoffmann TC, Mulrow CD, et al. The PRISMA 2020 statement: an updated guideline for reporting systematic reviews. BMJ 2021;372:n71. doi: 10.1136/bmj.n71. This work is licensed under CC BY 4.0. To view a copy of this license, visit <https://creativecommons.org/licenses/by/4.0/>

**Supporting Information 2 – Search strategy**

The key terms used in the search strategy included “front-of-pack nutrition labelling” and “sugar consumption”.

***Table S2.1. Search strategy used in Ovid Medline***

| ***Line Number*** | ***Search Term*** | ***Results*** |
| --- | --- | --- |
| 1 | ("food label*" or "nutrition* label").mp. | 5,749 |
| 2 | (front adj4 (pack* or "nutrition* label*")).mp. | 944 |
| 3 | FOPL.mp. | 107 |
| 4 | ("traffic light*” OR “red label" or "green label" or "Wheel of health" or "colored GDA" or "coloured GDA" or "5 CNL" or "Color Nutrition" or "colour nutrition" or "Nutriscore" or "Nutri-score" or "simplified nutrition labelling system" or "SENS" or "colour-coded" or "color-coded" or "colour coding" or "color coding" or "Evolved Nutrition Label" or "warning label" or "warning labels" or "stop sign warning*" or "health star?" or "reference intake" or "reference intakes" or keyhole or "choice logo" or "healthy choice" or "health tick*" or "heart symbol" or "guideline daily amount*" or "daily guideline intake*" or "reference intake*” OR “energy icon").mp. | 18,474 |
| 5 | ("nutrition* sign" or "nutrition* symbol" or "nutrition* tick" or "nutrition* rating*" or "nutrition* mark*" or "nutrition* logo*" or "nutrition* warning").mp. | 992 |
| 6 | 1 or 2 or 3 or 4 or 5 | 24,671 |
| 7 | (sugar* or sucrose or syrup* or beverage* or coke or cola or drink* or soda or sweet* or chocolate* or "ice cream" or "ice cream*" or candy or candies or cookie* or jam or confectioner* or confectionar* or cake* or jelly or jellies or pastries or pastry or biscuit* or bakery or bakeries or juice* or honey or pie or pies or dessert* or cocoa or "chewing gum" or "chewing gums" or liquorice* or marmalade* or "Sugar sweetened beverage" or "carbonated drink*" or "carbonated beverage*" or "soft drink*" or "fructose corn syrup" or cereal* or smoothie* or milkshake* or molasses or fructose or glucose or "discretionary food*" or snacks or "junk food*" or lolly or lollies or cariogenic or "dried fruit*" or "ultra-processed food*").mp. | 1,325,620 |
| 8 | exp Sugars/ | 463,144 |
| 9 | 7or 8 | 1,380,138 |
| 10 | 6 and 9 | 2,873 |
| 11 | limit 10 to (english language and yr="2005 -Current") | **2,424** |

**Note.** The search was conducted in the Ovid Medline database on 26 June 2024, yielding an initial 2,424 records.

***Table S2.2. Search strategy used in Web of Science***

| ***Line Number*** | ***Search Term*** | ***Results*** |
| --- | --- | --- |
| 1 | TS=(“food label*” or "nutrition* label*") | 5,648 |
| 2 | TS=(front NEAR/4 (pack* OR "nutrition* labeling")) | 1,976 |
| 3 | TS=(FOPL) | 146 |
| 4 | TS=("traffic light*" OR "red label" OR "green label" OR "Wheel of health" OR "colored GDA" OR "coloured GDA" OR "5 CNL" OR "Color Nutrition" OR "colour nutrition" OR "Nutriscore" OR "Nutri-score" OR "simplified nutrition labelling system" OR "SENS" OR "colour-coded" OR "color-coded" OR "colour coding" OR "color coding" OR "Evolved Nutrition Label" OR "warning label" OR "warning labels" OR "stop sign warning*" OR "health star$" OR "reference intake" OR "reference intakes" OR keyhole OR "choice logo" OR "healthy choice" OR "health tick*" OR "heart symbol" OR "guideline daily amount*" OR "daily guideline intake*" OR "reference intake*"OR "energy icon") | 32,317 |
| 5 | TS=("nutrition* sign" OR "nutrition* symbol" OR "nutrition* tick" OR "nutrition* rating*" OR "nutrition* mark*" OR "nutrition* logo*" OR "nutrition* warning") | 1,155 |
| 6 | 1 or 2 or 3 or 4 or 5 | 39,269 |
| 7 | TS=(sugar* OR sucrose OR syrup* OR beverage* OR coke OR cola OR drink* OR soda OR sweet* OR chocolate* OR "ice cream" OR "ice cream*" OR candy OR candies OR cookie* OR jam OR confectioner* OR confectionar* OR cake* OR jelly OR jellies OR pastries OR pastry OR biscuit* OR bakery OR bakeries OR juice* OR honey OR pie OR pies OR dessert* OR cocoa OR "chewing gum" OR "chewing gums" OR liquorice* OR marmalade* OR "Sugar sweetened beverage" OR "carbonated drink*" OR "carbonated beverage*" OR "soft drink*" OR "fructose corn syrup" OR cereal* OR smoothie* OR milkshake* OR molasses OR fructose OR glucose OR "discretionary food*" OR snacks OR "junk food*" OR lolly OR lollies OR cariogenic OR "dried fruit*" OR "ultra-processed food*") | 2,256,665 |
| 8 | 6 AND 7 | 3,569 |
| 9 | PY=(2005-2024) AND 8 AND (LA==("ENGLISH")) | **3,034** |

**Note.** The search was conducted in the Web of Science database on 26 June 2024, yielding an initial 3,034 records.

***Table S2.3. Search strategy used in Scopus***

| ***Line Number*** | ***Search Term*** | ***Results*** |
| --- | --- | --- |
| 1 | TITLE-ABS-KEY ( "food label*" OR "nutrition* label" ) | 8,336 |
| 2 | TITLE-ABS-KEY ( front W/4 ( pack* OR "nutrition* label*" ) ) | 2,227 |
| 3 | TITLE-ABS-KEY ( FOPL ) | 176 |
| 4 | TITLE-ABS-KEY ( "traffic light*" OR "red label" OR "green label" OR "wheel of health" OR "colored gda" OR "coloured gda" OR "5 cnl" OR "color nutrition" OR "colour nutrition" OR "nutriscore" OR "nutri-score" OR "simplified nutrition labelling system" OR "sens" OR "colour-coded" OR "color-coded" OR "colour coding" OR "color coding" OR "evolved nutrition label" OR "warning label" OR "warning labels" OR "stop sign warning*" OR "health star?" OR "reference intake" OR "reference intakes" OR keyhole OR "choice logo" OR "healthy choice" OR "health tick*" OR "heart symbol" OR "guideline daily amount*" OR "daily guideline intake*" OR "reference intake*" OR "energy icon" ) | 72,133 |
| 5 | TITLE-ABS-KEY ( "nutrition* sign" OR "nutrition* symbol" OR "nutrition* tick" OR "nutrition* rating*" OR "nutrition* mark*" OR "nutrition* logo*" OR "nutrition* warning" ) | 1,242 |
| 6 | 1 or 2 or 3 or 4 or 5 | 81,877 |
| 7 | TITLE-ABS-KEY ( sugar* OR sucrose OR syrup* OR beverage* OR coke OR cola OR drink* OR soda OR sweet* OR chocolate* OR "ice cream*" OR candy OR candies OR cookie* OR jam OR confectioner* OR confectionar* OR cake* OR jelly OR jellies OR pastries OR pastry OR biscuit* OR bakery OR bakeries OR juice* OR honey OR pie OR pies OR dessert* OR cocoa OR "chewing gum*" OR liquorice* OR marmalade* OR "Sugar sweetened beverage" OR "carbonated drink*" OR "carbonated beverage*" OR "soft drink*" OR "fructose corn syrup" OR cereal* OR smoothie* OR milkshake* OR molasses OR fructose OR glucose OR "discretionary food*" OR snacks OR "junk food*" OR lolly OR lollies OR cariogenic OR "dried fruit*" OR "ultra-processed food*" ) | 2,506,172 |
| 8 |  | 6,532 |
| 9 | PUBYEAR > 2004 AND PUBYEAR < 2025 AND ( LIMIT-TO ( LANGUAGE , "English" ) ) | **5,554** |

**Note.** The search was conducted in the Scopus database on 26 June 2024, yielding an initial 5,554 records.

***Table S2.4. Search strategy used in EMBASE***

| ***Line Number*** | ***Search Term*** | ***Results*** |
| --- | --- | --- |
| 1 | ("food label*" or "nutrition* label").mp. | 3,096 |
| 2 | (front adj4 (pack* or "nutrition* label*")).mp. | 1,145 |
| 3 | FOPL.mp. | 126 |
| 4 | ("traffic light*” OR “red label" or "green label" or "Wheel of health" or "colored GDA" or "coloured GDA" or "5 CNL" or "Color Nutrition" or "colour nutrition" or "Nutriscore" or "Nutri-score" or "simplified nutrition labelling system" or "SENS" or "colour-coded" or "color-coded" or "colour coding" or "color coding" or "Evolved Nutrition Label" or "warning label" or "warning labels" or "stop sign warning*" or "health star?" or "reference intake" or "reference intakes" or keyhole or "choice logo" or "healthy choice" or "health tick*" or "heart symbol" or "guideline daily amount*" or "daily guideline intake*" or "reference intake*” OR “energy icon").mp. | 29,347 |
| 5 | ("nutrition* sign" or "nutrition* symbol" or "nutrition* tick" or "nutrition* rating*" or "nutrition* mark*" or "nutrition* logo*" or "nutrition* warning").mp. | 1,512 |
| 6 | 1 or 2 or 3 or 4 or 5 | 3,387 |
| 7 | (sugar* or sucrose or syrup* or beverage* or coke or cola or drink* or soda or sweet* or chocolate* or "ice cream" or "ice cream*" or candy or candies or cookie* or jam or confectioner* or confectionar* or cake* or jelly or jellies or pastries or pastry or biscuit* or bakery or bakeries or juice* or honey or pie or pies or dessert* or cocoa or "chewing gum" or "chewing gums" or liquorice* or marmalade* or "Sugar sweetened beverage" or "carbonated drink*" or "carbonated beverage*" or "soft drink*" or "fructose corn syrup" or cereal* or smoothie* or milkshake* or molasses or fructose or glucose or "discretionary food*" or snacks or "junk food*" or lolly or lollies or cariogenic or "dried fruit*" or "ultra-processed food*").mp. | 1,923,722 |
| 8 | exp Sugars/ | 1,282,439 |
| 9 | 7or 8 | 2,453,895 |
| 10 | 6 and 9 | 4,855 |
| 11 | limit 10 to (english language and yr="2005 -Current") | **4,257** |

**Note.** The search was conducted in the EMBASE database on 26 June 2024, yielding an initial 4,257 records.

**Supporting Information 3 – Eligible criteria**

***Table S3. Eligible criteria***

| Parameter | Inclusion criteria | Exclusion criteria |
| --- | --- | --- |
| Population | Whole population | Studies focus on specific clinical groups/people with specific medical condition/ people stay in institutional settings. |
| Intervention | Any format of front-of-pack nutrition labelling implemented in a real-life setting, or an experimental setting  No restriction for food retail settings | - Studies where a combination of front-of pack nutrition labelling intervention and other intervention, for example, health promotion on free sugar consumption - Exposed to additional information - Back-of-pack nutrition labelling intervention |
| Comparator | Any other preventive intervention or no intervention | No comparison |
| Outcome | - Change in purchasing behaviour/sugar intake/ the sales of food and drinks with high free sugar/ sugar consumption before and after the implementation of front-of-nutrition food labelling, or between groups exposed to front-of-pack food labelling and those not exposed, stratified by SES. SES is defined as individual measures of income, education, occupation, and social class or area-level deprivation, such as SES index according to the area postcode. - The outcome can be reported in either absolute or relative scale. | - The purchasing behaviour/sugar intake/the sales is not stratified by SES before and after the implementation of front-of-pack nutrition labelling. - The purchasing behaviour/sugar intake/the sales is not stratified by SES between groups exposed to front-of-pack nutrition labelling and those not exposed. - Purchasing behaviour is for food supply. |
| Time | Published since 2005 to current | Published before 2005 |
| Geography | All | No restriction |
| Language | Restricted to English language publications. | Non-English language publications |
| Study design | Empirical studies (RCT and non-RCT) and observational studies (cross-sectional studies, cohort studies, natural experiments, and ecological studies), and simulation studies. | Any reviews including systematic review, scoping review, and meta-analysis, qualitative study, conference abstracts |

**Supporting Information 4 – CHEERS 2022 Checklist**

***Table S4.1. Assessment of Reporting Quality Using the CHEERS 2020 Checklist: Grummon et al. 2019***

| **Topic** | **No.** | **Item** | **Location where item is reported** |
| --- | --- | --- | --- |
| **Title** |  |  |  |
|  | 1 | Identify the study as an economic evaluation and specify the interventions being compared. | Title, page 1; Identification of the study as an economic evaluation--NR |
| **Abstract** |  |  |  |
|  | 2 | Provide a structured summary that highlights context, key methods, results, and alternative analyses. | Abstract, page 1 |
| **Introduction** |  |  |  |
| **Background and objectives** | 3 | Give the context for the study, the study question, and its practical relevance for decision making in policy or practice. | Introduction, last paragraph |
| **Methods** |  |  |  |
| **Health economic analysis plan** | 4 | Indicate whether a health economic analysis plan was developed and where available. | Methods, 2 line |
| **Study population** | 5 | Describe characteristics of the study population (such as age range, demographics, socioeconomic, or clinical characteristics). | Method, study population section |
| **Setting and location** | 6 | Provide relevant contextual information that may influence findings. | Method, study design and population section |
| **Comparators** | 7 | Describe the interventions or strategies being compared and why chosen. | Method, Food replacement scenarios section |
| **Perspective** | 8 | State the perspective(s) adopted by the study and why chosen. | NR |
| **Time horizon** | 9 | State the time horizon for the study and why appropriate. | Methods, procedures section |
| **Discount rate** | 10 | Report the discount rate(s) and reason chosen. | Not sure |
| **Selection of outcomes** | 11 | Describe what outcomes were used as the measure(s) of benefit(s) and harm(s). | Methods, procedures section |
| **Measurement of outcomes** | 12 | Describe how outcomes used to capture benefit(s) and harm(s) were measured. | Methods, procedures section |
| **Valuation of outcomes** | 13 | Describe the population and methods used to measure and value outcomes. | Methods, statistical analysis |
| **Measurement and valuation of resources and costs** | 14 | Describe how costs were valued. | NR |
| **Currency, price date, and conversion** | 15 | Report the dates of the estimated resource quantities and unit costs, plus the currency and year of conversion. | NR |
| **Rationale and description of model** | 16 | If modelling is used, describe in detail and why used. Report if the model is publicly available and where it can be accessed. | Methods, statistical analysis |
| **Analytics and assumptions** | 17 | Describe any methods for analysing or statistically transforming data, any extrapolation methods, and approaches for validating any model used. | Methods, procedures section |
| **Characterising heterogeneity** | 18 | Describe any methods used for estimating how the results of the study vary for subgroups. | Methods, statistical analysis |
| **Characterising distributional effects** | 19 | Describe how impacts are distributed across different individuals or adjustments made to reflect priority populations. | Methods, statistical analysis |
| **Characterising uncertainty** | 20 | Describe methods to characterise any sources of uncertainty in the analysis. | Appendix, uncertainty analyses section |
| **Approach to engagement with patients and others affected by the study** | 21 | Describe any approaches to engage patients or service recipients, the general public, communities, or stakeholders (such as clinicians or payers) in the design of the study. | NR |
| **Results** |  |  |  |
| **Study parameters** | 22 | Report all analytic inputs (such as values, ranges, references) including uncertainty or distributional assumptions. | Results |
| **Summary of main results** | 23 | Report the mean values for the main categories of costs and outcomes of interest and summarise them in the most appropriate overall measure. | Outcome of interest--Results; cost-- NR |
| **Effect of uncertainty** | 24 | Describe how uncertainty about analytic judgments, inputs, or projections affect findings. Report the effect of choice of discount rate and time horizon, if applicable. | Results |
| **Effect of engagement with patients and others affected by the study** | 25 | Report on any difference patient/service recipient, general public, community, or stakeholder involvement made to the approach or findings of the study | NR |
| **Discussion** |  |  |  |
| **Study findings, limitations, generalisability, and current knowledge** | 26 | Report key findings, limitations, ethical or equity considerations not captured, and how these could affect patients, policy, or practice. | Discussion |
| **Other relevant information** |  |  |  |
| **Source of funding** | 27 | Describe how the study was funded and any role of the funder in the identification, design, conduct, and reporting of the analysis | Acknowledgements, 3 paragraph |
| **Conflicts of interest** | 28 | Report authors conflicts of interest according to journal or International Committee of Medical Journal Editors requirements. | Acknowledgements, last paragraph |

***Table S4.2. Assessment of Reporting Quality Using the CHEERS 2020 Checklist: Wanseliu et al. 2022***

| **Topic** | **No.** | **Item** | **Location where item is reported** |
| --- | --- | --- | --- |
| **Title** |  |  |  |
|  | 1 | Identify the study as an economic evaluation and specify the interventions being compared. | Title, page 1 |
| **Abstract** |  |  |  |
|  | 2 | Provide a structured summary that highlights context, key methods, results, and alternative analyses. | Abstract, page 1 |
| **Introduction** |  |  |  |
| **Background and objectives** | 3 | Give the context for the study, the study question, and its practical relevance for decision making in policy or practice. | Introduction, last paragraph |
| **Methods** |  |  |  |
| **Health economic analysis plan** | 4 | Indicate whether a health economic analysis plan was developed and where available. | NR |
| **Study population** | 5 | Describe characteristics of the study population (such as age range, demographics, socioeconomic, or clinical characteristics). | Method, study design and population section |
| **Setting and location** | 6 | Provide relevant contextual information that may influence findings. | Method, study design and population section |
| **Comparators** | 7 | Describe the interventions or strategies being compared and why chosen. | Method, Food replacement scenarios section |
| **Perspective** | 8 | State the perspective(s) adopted by the study and why chosen. | NR |
| **Time horizon** | 9 | State the time horizon for the study and why appropriate. | NR |
| **Discount rate** | 10 | Report the discount rate(s) and reason chosen. | Not sure |
| **Selection of outcomes** | 11 | Describe what outcomes were used as the measure(s) of benefit(s) and harm(s). | Methods, Food replacement scenarios section |
| **Measurement of outcomes** | 12 | Describe how outcomes used to capture benefit(s) and harm(s) were measured. | Methods, Food replacement scenarios section |
| **Valuation of outcomes** | 13 | Describe the population and methods used to measure and value outcomes. | Methods, statistical methods |
| **Measurement and valuation of resources and costs** | 14 | Describe how costs were valued. | not sure |
| **Currency, price date, and conversion** | 15 | Report the dates of the estimated resource quantities and unit costs, plus the currency and year of conversion. | NR |
| **Rationale and description of model** | 16 | If modelling is used, describe in detail and why used. Report if the model is publicly available and where it can be accessed. | NA |
| **Analytics and assumptions** | 17 | Describe any methods for analysing or statistically transforming data, any extrapolation methods, and approaches for validating any model used. | Method, statistical methods section |
| **Characterising heterogeneity** | 18 | Describe any methods used for estimating how the results of the study vary for subgroups. | Method, statistical methods section |
| **Characterising distributional effects** | 19 | Describe how impacts are distributed across different individuals or adjustments made to reflect priority populations. | Method, statistical methods section |
| **Characterising uncertainty** | 20 | Describe methods to characterise any sources of uncertainty in the analysis. | NR |
| **Approach to engagement with patients and others affected by the study** | 21 | Describe any approaches to engage patients or service recipients, the general public, communities, or stakeholders (such as clinicians or payers) in the design of the study. | NR |
| **Results** |  |  |  |
| **Study parameters** | 22 | Report all analytic inputs (such as values, ranges, references) including uncertainty or distributional assumptions. | NR |
| **Summary of main results** | 23 | Report the mean values for the main categories of costs and outcomes of interest and summarise them in the most appropriate overall measure. | NR--reported median |
| **Effect of uncertainty** | 24 | Describe how uncertainty about analytic judgments, inputs, or projections affect findings. Report the effect of choice of discount rate and time horizon, if applicable. | NR |
| **Effect of engagement with patients and others affected by the study** | 25 | Report on any difference patient/service recipient, general public, community, or stakeholder involvement made to the approach or findings of the study | NR |
| **Discussion** |  |  |  |
| **Study findings, limitations, generalisability, and current knowledge** | 26 | Report key findings, limitations, ethical or equity considerations not captured, and how these could affect patients, policy, or practice. | Discussion |
| **Other relevant information** |  |  |  |
| **Source of funding** | 27 | Describe how the study was funded and any role of the funder in the identification, design, conduct, and reporting of the analysis | Acknowledgements, 5 line |
| **Conflicts of interest** | 28 | Report authors conflicts of interest according to journal or International Committee of Medical Journal Editors requirements. | Acknowledgements, 11 line |
